# Enhanced Permeation of an Antiemetic Drug from Buccoadhesive Tablets by Using Bile Salts as Permeation Enhancers: Formulation Characterization, *In Vitro*, and *Ex Vivo* Studies

**DOI:** 10.3797/scipharm.1505-15

**Published:** 2015-07-29

**Authors:** C. P. Jain, Garima Joshi, Udichi Kataria, Komal Patel

**Affiliations:** Department of Pharmaceutical Sciences, Mohanlal Sukhadia University, Main University Campus, Udaipur, Rajasthan, 313001, India

**Keywords:** Buccal tablet, Ex vivo, Antiemetic, Prochlorperazine, Bile salts

## Abstract

Buccal bioadhesive bilayer tablets of prochlorperazine maleate were designed and formulated by using buccoadhesive polymers such as hydroxypropylmethyl cellulose, Carbopol 934P, and sodium alginate. Physicochemical characteristics like the uniformity of weight, hardness, thickness, surface pH, drug content, swelling index, microenvironment pH, *in vitro* drug release, and *in vivo* buccoadhesion time of the prepared tablets were found to be dependent on the type and composition of the buccoadhesive materials used. The effect of bile salts on the permeation was studied through porcine buccal mucosa and it was found that out of three bile salts incorporated (sodium glycholate, sodium taurocholate, and sodium deoxycholate), sodium glycholate enhanced the permeation rate of prochlorperazine maleate by an enhancement factor of 1.37.

## Introduction

Although the pharmaceutical world is full of research and discoveries in many novel and advanced drug delivery systems for therapeutic use, the popularity of oral dosage forms, particularly tablets, have not been eclipsed. This is due to its various advantages and economical reasons, but the major drawbacks of tablets are the need to be swallowed, gastrointestinal degradation, and the first-pass effect in many cases.

The buccal route is a subject of growing interest because of its numerous advantages [1, 2]. It is well-known that the absorption of therapeutic compounds from the oral mucosa provides direct entry of the drug into systemic circulation, avoiding first-pass hepatic metabolism and gastrointestinal drug degradation and thus, improves bioavailability. In addition, it is robust, easily accessible, and shows high patient compliance [3]. It is safe since the device can be easily administered and even removed from the site of application, stopping the input of drug whenever desired.

A variety of drugs are reported to be absorbed through the oral mucosa following administration as solutions, conventional tablets, or capsules. However, the conventional buccal dosage form shows two main limitations: (i) due to involuntary swallowing of the dosage form or the drug due to the salivary flow, an important part of the drug may not be available for absorption and (ii) buccal dosage forms do not allow for drinking and eating. Therefore, their administration is restricted to short periods of time. From a technological point of view, an ideal buccal dosage form must have following properties: (i) it must maintain its position in the mouth for a few hours, (ii) release the drug in a controlled fashion within a short period of time, and (iii) provide drug release in a unidirectional way towards the mucosa. Considering the first requirement, strong adhesive contact to the mucosa has to be established by using mucoadhesive polymers as excipients. If these mucoadhesive excipients are able to control drug release, the second requirement will also be achieved. The third objective can be fulfilled using bilayered devices [2, 3]. Therefore, the first step in the development of a buccal device is the selection of an appropriate adhesive. Many bioadhesive polymers have been investigated for the fabrication of buccal devices including carbopol, polycarbophil, and the cellulose derivatives sodium carboxymethylcellulose, hydroxypropylcellulose, as well as hydroxypropyl methylcellulose [4–6]. When hydrated with water, these polymers adhere to the oral mucosa, withstanding salivation, tongue movements, and swallowing for a significant period of time. Bioadhesive polymers have extensively been employed in buccal drug delivery systems in the form of adhesive patches, adhesive films, adhesive tablets, and buccal gels. Drugs with slow or incomplete permeation can be administered through the buccal route by using penetration enhancers with the drug [7–10].

Antiemetic agents are used to suppress nausea and vomiting. They are widely used in motion sickness, drug-induced and post-anesthetic nausea and vomiting diseases, migraines, gastroenteritis-induced nausea and vomiting, malignancy- and cancer chemotherapy-related vomiting, morning sickness, etc. The oral route of administration of antiemetics is impractical for patients who are vomiting or suffer from impaired gastric emptying. For drugs with low bioavailability, partial drug loss by emesis will result in therapeutic failure too. Thus, the buccal drug delivery system for an antiemetic agent can be most suitable for patients suffering from motion sickness with no access to water. Buccal delivery of antiemetics offers advantages such as ease of administration, painless noninvasive therapy, and rapid onset of action.

Prochlorperazine maleate has been accepted as an effective antiemetic for more than 50 years; however, its therapeutic success has been limited by its low and variable absorption rate and high first-pass metabolism [11]. Thus, bioadhesive buccal tablets appear to be suitable dosage forms for the delivery of prochlorperazine maleate and necessitate the use of mucoadhesive polymers to prolong the residence time of the dosage form on the absorptive membrane.

The objective of the present study was to formulate buccal mucoadhesive bilayered tablets of prochlorperazine maleate to improve the bioavailability and prolong the residence time by using Carbopol and HPMC K4M as mucoadhesive polymers, ensuring the drug release in a unidirectional way to the mucosa, thus avoiding the loss of drug due to washout with saliva. Additionally, the effect of various bile salts like sodium glycholate, sodium taurocholate, and sodium deoxycholate on the permeation rate was studied.

## Results and Discussion

### Physicochemical Characterization of Bilayered Bioadhesive Tablets of the Trial Series

The results of the physicochemical characteristics like uniformity of weight, hardness, thickness, surface pH, and drug content are shown in Table 3. The maximum and minimum average weight of the tablet was found to be 90±0.2 mg and 89.3±0.57 mg, respectively. As none of the formulations showed a standard deviation of more than ±10% (I.P. limit) for any of the tablets tested, the prepared formulation complied with the weight variation test. The hardness of the adhesive layer of all formulations was between 2.64±0.134 to 3.06±0.14 kp while the total hardness of the bilayer tablet was between 6.08±0.22 to 5.82±0.14 kp for all the formulations. The maximum and minimum average thicknesses of the formulation were found to be 1.5±.02 mm and 1.42±.01 mm, resulting in 1.42 mm on average.

**Tab. 1:**
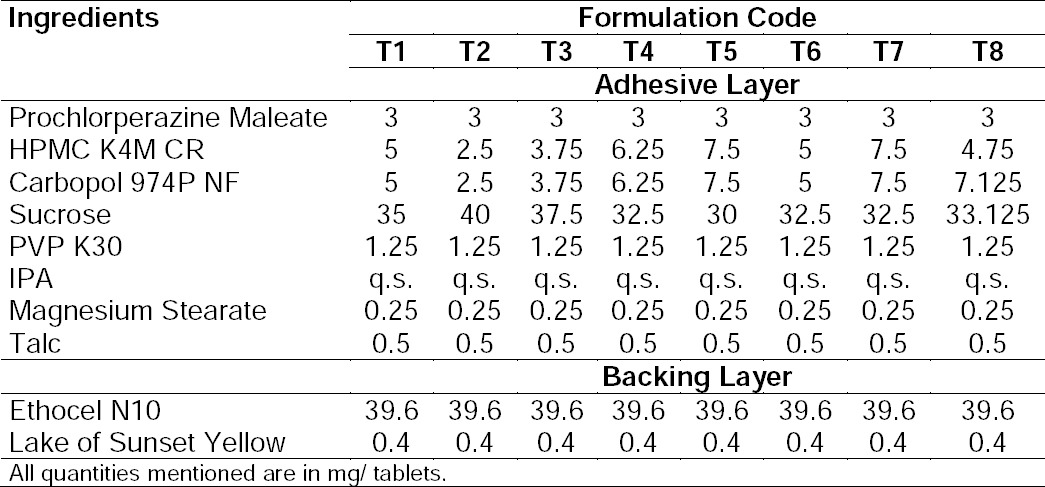
Composition of the buccal tablets of trial series

**Tab. 2:**
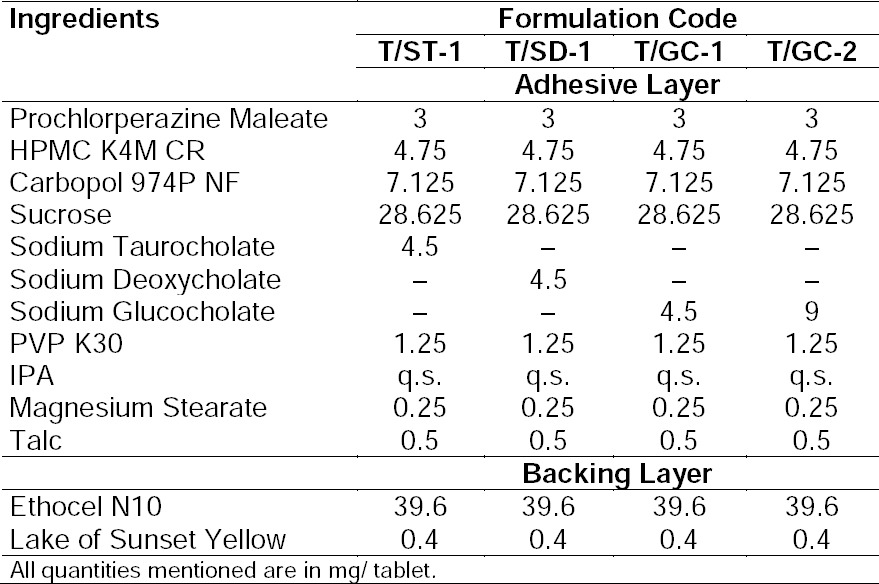
Composition of the buccal tablets containing bile salts

**Tab. 3:**
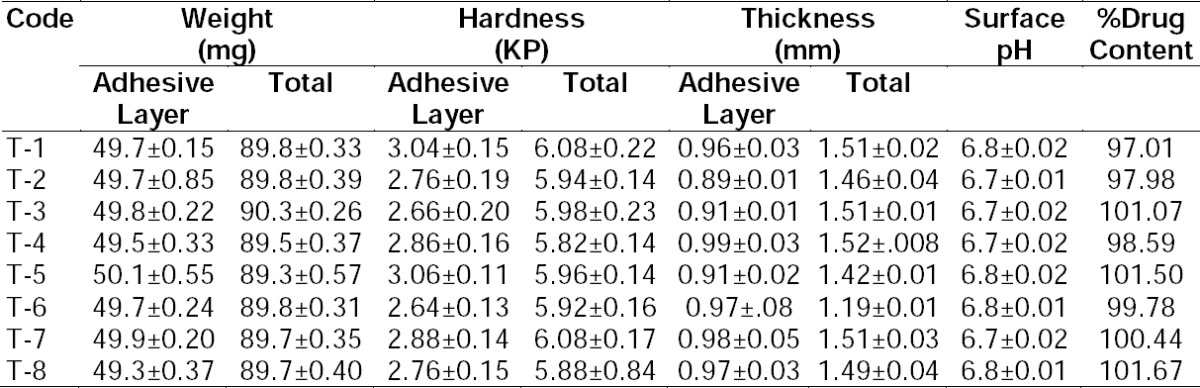
Physicochemical characterization of the buccal tablets of trial series

The pH of the microenvironment around the surface of the formulation of the trial series was close to 7.0 and hence, no irritation to the mucosal surface was expected. The maximum and minimum drug content for all formulations was found to be 97.01% and 101.67%, respectively. All the results of physicochemical parameters were within the limits specified by I.P.

**Fig. 1:**
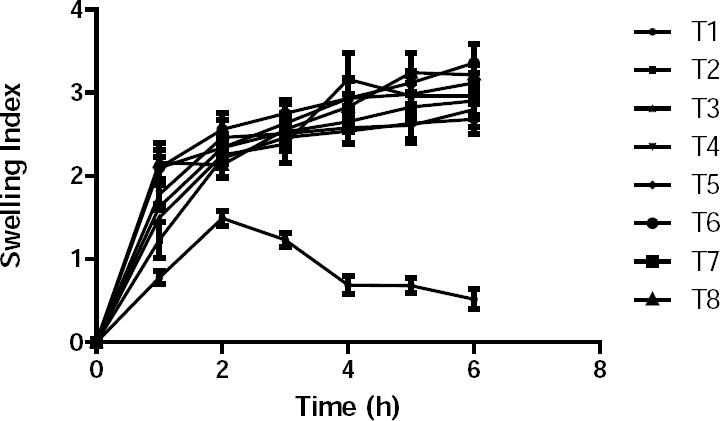
Swelling index for the trial batch series of buccal bilayered tablets.

The swelling index (Figure 1) revealed that the hydration rate and water uptake of the formulation increased with increasing amounts of the polymer combination. The swelling index of formulations T2 and T3 decreased after 2 h and 5 h, respectively, due to low polymer concentration. Hydration rate and water uptake of the remaining formulations were almost similar, indicating that there was no major effect on the swelling index for formulations with a polymer content >30%.

**Fig. 2:**
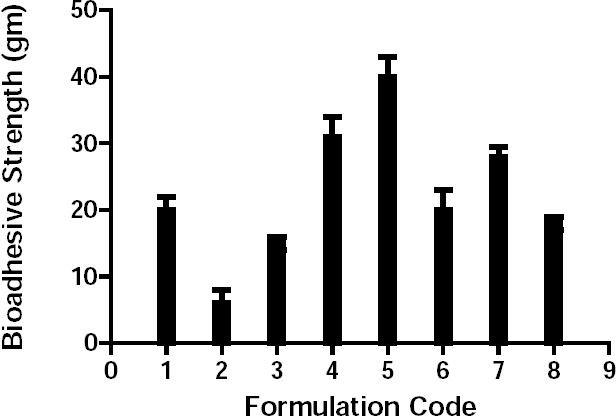
Bioadhesive strength of the trial batches of buccal bilayered tablets.

The bioadhesive strength of the formulation is a function of the polymer nature, viscosity, and surface area of the tablet. The bioadhesive strength increased with the polymer content (Figure 2). Thus, the bioadhesive strength of formulation T5 was the highest, whereas that of formulation T2 was the lowest due to the low polymer concentration.

**Fig. 3:**
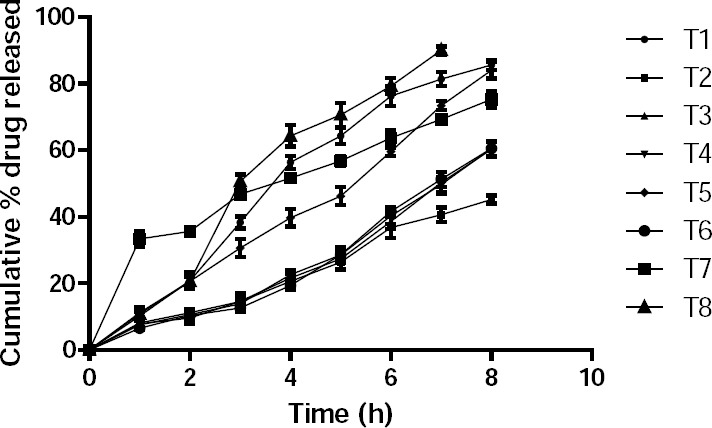
Cumulative % drug release of the trial batches of buccal bilayered tablets.

The plot of cumulative drug release vs. time of the formulations is shown in Figure 3. It reveals that the cumulative drug release is the function of optimum polymer concentration.

Drug release after 8 hours was 90.02% for formulation T1 with a polymer concentration of 20% by weight, whereas that of formulations T2, T3, T4, T5 with polymer concentrations of 10%, 15%, 25%, and 30% were 54.03%, 63.91%, 79.35%, and 74.98%, respectively. This means that drug release from the formulation was fast at the optimum concentration (20%), whereas below and above, drug release was delayed. Furthermore, formulation T6 with an altered polymer ratio showed 91.92% drug release after 8 hours in a controlled manner, while formulation T8 showed 99.87% drug release already after 6 hours. Thus, T8 showed maximum drug release within a minimum of time.

### Selection of Optimized Formulation for Further Studies

The formulation of bioadhesive bilayered tablets of prochlorperazine maleate for further studies was selected according to the results of adhesive strength, swelling index, surface pH, and *in vitro* drug release profiles.

Formulation T8, having a polymer combination of 4.75 mg of HPMC K4M and 7.125 mg of Carbopol 974P NF, was characterized by an adhesive strength of 18.836, swelling index of 3.246 after 5 hours, and *in vitro* release of 99.87% drug release within 5 hours. Thus, formulation T8 had sufficient bioadhesive strength to remain in place till all the drug was released and was selected for further studies.

### Characterization of Bilayered Bioadhesive Tablets Containing Bile Salts

The results of physiochemical characteristics like uniformity of weight, hardness, thickness, surface pH, and drug content are listed in Table 4. The maximum percentage deviation was found to be 1.172% from all the formulation containing bile salts. All the formulations complied with the I.P. specifications. The hardness of the adhesive layers of all formulations was between 2.678±0.081 to 3.06±0.114 kp while the total hardness of the bilayered tablets was in the range of 5.99±0.468 to 6.388±0.187 kp for all formulations. The maximum and minimum average thickness of all the formulations was found to be 1.55 and 1.42 mm, respectively.

**Tab. 4:**
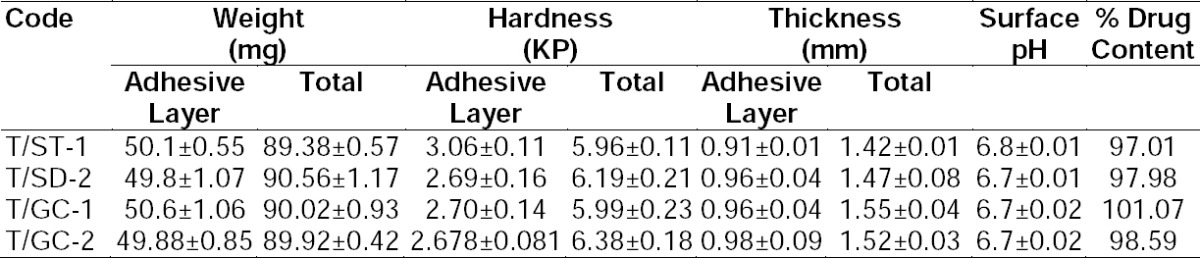
Physicochemical characterization of the buccal tablets containing bile salts

The drug content of all the formulations was between 97.01% and 101.07%, complying with the limits specified by the I.P. The pH of the microenvironment around the surface of the formulation was near to the neutral value of 7 and hence, was without any irritation of the mucosal surface where it was applied.

**Fig. 4:**
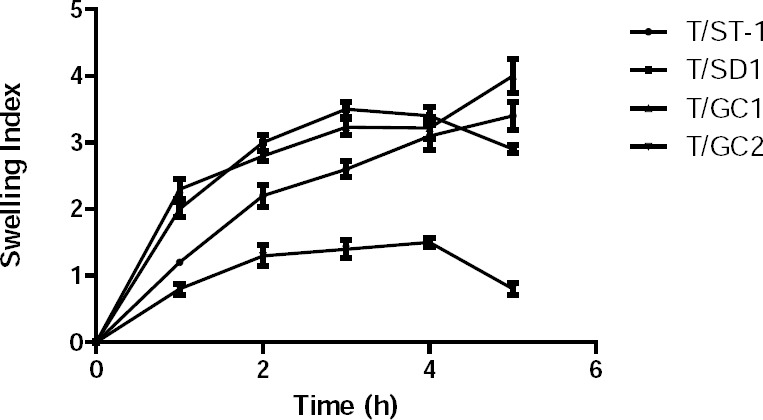
Swelling index of the buccal bilayered tablets containing bile salts.

There was a remarkable difference in the swelling index (Figure 4) of the formulations containing bile salts compared to the control, T8. The swelling index of the formulation containing sodium deoxycholate was lower, while that of the formulation containing sodium glycholate was higher. No remarkable alteration of the swelling index was observed in the case of sodium taurocholate.

**Fig. 5:**
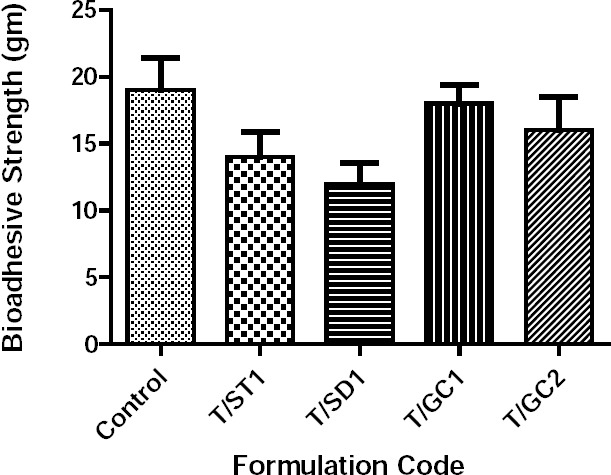
Bioadhesive strength of the buccal bilayered tablets containing bile salts.

**Fig. 6:**
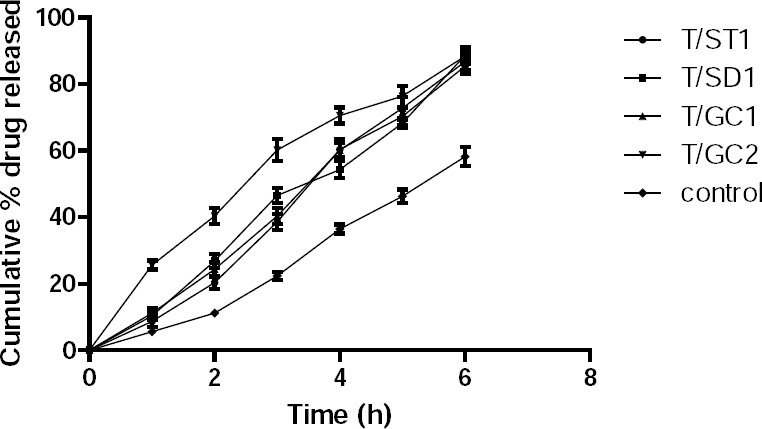
Cumulative % drug release of buccal bilayered tablets containing bile salts and marketed conventional tablet (control).

The bioadhesive strength (Figure 5) of formulation T/GC-1 was highest among all of the formulations containing bile salts, which was slightly lower than that of the control, T8. Additionally, *in vitro* release studies (Figure 6) were performed in comparison with a marketed conventional tablet (control). After 6 hours, the amount of drug released from the buccal tablets containing bile salts was significantly faster compared with the marketed tablet. However, a similar amount of drug was released after 6 hours from all of the tablets with bile salts, which revealed that an increasing content of surfactants would be without effect.

**Fig. 7:**
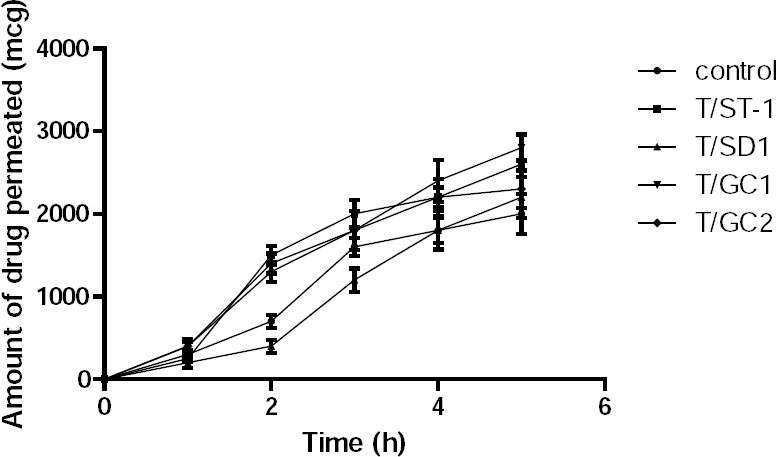
Amount of the drug permeated through the buccal bilayered tablets containing bile salts and the control (T8 optimized buccal tablet without bile salts).

The results of the permeation studies (Figure 7 and Table 5) indicated that prochlorperazine maleate from the control formulation permeated at a steady a flux of 3.01 which improved considerably to 3.7606 and 3.757 for formulations containing sodium glycolcholate. Furthermore, the flux of prochlorperazine maleate from formulations containing sodium taurocholate increased to 3.65, whereas the effect of sodium deoxycholate was lower than that of glycocholate and taurocholate. Thus, the incorporation of sodium glycocholate improved the permeation of prochlorperazine maleate by an enhancement factor of 1.37. Furthermore, increasing the sodium glycholate content from 5% to 15% did not remarkably increase the permeation of prochlorperazine maleate. Thus, the enhancement of permeation of prochlorperazine maleate by the different bile salts tested was found to follow the order: sodium glycholate > sodium taurocholate > sodium deoxycholate.

**Tab. 5:**
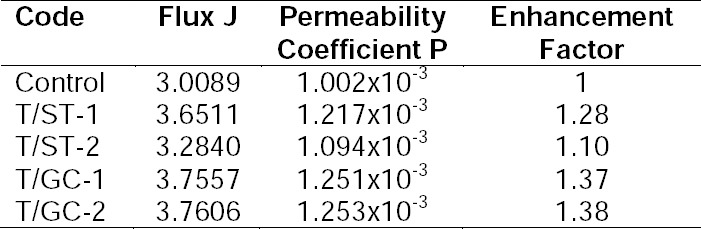
Permeation parameters of prochlorperazine maleate

## Conclusion

Buccal bilayered tablets of prochlorperazine maleate were formulated using bile salts and were evaluated considering physicochemical parameters as well as swelling index, bioadhesive strength, and *in vitro* and *ex vivo* drug release. Among the formulations containing bile salts, a remarkable increase in the flux of prochlorperazine maleate through porcine buccal mucosa was observed in formulations containing sodium glycocholate, followed by sodium taurocholate. There was no considerable increase in the flux in the presence of sodium deoxycholate. Formulations containing sodium glycocholate did not considerably improve bioadhesive strength, but increased the swelling index. The surface pH of all the formulations was within the tolerance limits of the buccal mucosa. Therefore, a buccal tablet of prochlorperazine maleate can be an effective substitute for the marketed preparation to provide a rapid onset of action as well as bypass hepatic first-pass. However, further animal studies are required for assessing safety in human beings.

## Experimental

### Materials

Prochlorperazine maleate was kindly provided by Amol Drug Pharma Ltd., India. HPMC K4M CR, Ethocel N-10, and Lake of Sunset Yellow were gift samples from Coloron Asia Ltd., Goa, India. Sodium taurocholate, sodium glucocholate, and sodium deoxycholate were supplied by Proditti Chemici, Italy. All other materials used in this study were of analytical grade.

### Preparation of Mucoadhesive Buccal Tablets

Mucoadhesive bilayered tablets (consisting of a backing layer and adhesive: drug reservoir layer) were made by covering one side with an inert ethylcellulose backing layer. Ethylcellulose was selected as a backing material because this hydrophobic polymer has very low water permeability, thus providing an impermeable backing layer that prevents drug loss [12].

The tablets were prepared by method given below involving following consecutive steps.

- To prepare the blend for the adhesive layer: all the mucoadhesive polymers were sieved #40, weighed, and blended together using PVP K-30 and non-aqueous medium in a glass mortar. Then the wet mass obtained was dried at 45°C in a tray dryer until the loss on weight of granules was below 1.5% w/w. The dried mass was then sifted and the granules obtained were lubricated with magnesium stearate and talc.
- To prepare the blend for the backing layer: Ethocel N-10 was mixed with the Lake of Sunset Yellow dye.
- Compression of the bilayered buccal tablets: 50 mg of the adhesive layer blend and 40 mg of the backing layer blend were weighed individually. The adhesive layer was compressed in a 12-station rotary compression machine using 8 mm (13/32 inches) flat surface punches to obtain a hardness of 2.5 to 3 kp. The backing layer was then added onto the primarily compressed adhesive layer and compressed to obtain a hardness of 5 to 6 kp. The bilayered tablets were prepared using the composition of ingredients as given in Table 1 and Table 2. The prepared tablets were evaluated for the following parameters:

#### Uniformity of Weight

The uniformity of weight was determined according to the specifications given in the Indian Pharmacopeia. The individual weights of the 10 bilayered tablets and compressed adhesive layer from each batch were determined accurately using an electronic balance (Schimadzu, Aux*220, Japan) and the sample mean and standard deviation of each batch were calculated. All of the experiments were performed in triplicate and the data are presented as mean ± SD.

#### Hardness

The hardness of the adhesive layer and bilayered tablet was conducted on 10 tablets from each batch using Hardness Tester 8M (Dr. Schleunger) and the average values were calculated.

#### Thickness

Thickness was measured using Digital Vernier Calipers. Ten tablets were selected at random from each batch and the crown-to-crown thickness of the bilayered tablet was measured. Similarly, the thickness of the adhesive layer was also measured.

#### Uniformity of the Drug Content

All solutions were protected from light throughout the test. One tablet was selected at random and crushed in a mortar and extracted in three quantities each of 10 ml of ethanol containing 1% v/v of strong ammonia solution. The extract was filtered and to the combined extracts, sufficient ethanol was added to produce 100 ml. Ten ml of this solution was diluted to 50 ml with ethanol and the absorbance of the resulting solution was measured at 255 nm.

#### Swelling Index

Bilayered tablets were weighed individually; the initial weight was considered as W_1_ and placed separately in Petri dishes containing 15 ml of phosphate buffer (pH 6.8) solution in such a way that the side of the tablet which attaches to the buccal membrane was positioned to the bottom of the Petri dishes with the backing membrane viewable from the top. Tablets were soaked in such a way that the core tablet was completely immersed in the buffer solution. At regular intervals until 4 h, the buccal tablets were removed from the Petri dish, and excess surface water was removed carefully using Whatman filter paper. The swollen tablets were then reweighed (W_2_). The experiment was repeated three times and the average W_1_ and W_2_ are reported. The swelling index (degree of swelling) was calculated using the formula given in the equation.

Degree of swelling = (W_2_ − W_1_)/W_1_

#### Surface pH

The method adopted by Bottenberg *et al*. [15] was used to determine the surface pH of the tablet. A combined pH meter glass electrode was used for this purpose. The bioadhesive tablets (n=3) were allowed to swell by keeping it in contact with 1 ml of distilled water for 2 hours at room temperature. The pH was measured by bringing the pH meter glass electrode (Cyber Ph-141, Cyberlab, India) in contact with the surface of the tablet and allowing it to equilibrate for 1 minute.

#### Ex Vivo Mucoadhesive Strength

Animal experiments were approved by the Institutional Animal Ethics Committee of Mohanlal Sukhadia University, Udaipur. The mucoadhesive strength of the buccal tablet was measured using a modified physical balance method.

Porcine buccal mucosa was used as a model membrane and phosphate buffer pH 6.8 was used as moistening fluid. The porcine buccal mucosa was obtained from a local slaughter house and kept in a Krebs buffer at 4°C during transportation. The underlying mucous membrane was separated using a surgical blade and was washed thoroughly with phosphate buffer of pH 6.8. It was then secured firmly on the Teflon using a rubber band and placed in a beaker filled with phosphate buffer of pH 6.8 up to the upper surface of the buccal mucosa to maintain its viability throughout the experiment.

The preload of 5 g was placed on the right pan for 5 min (preload) to establish adhesion bonding between the bilayer tablet and porcine buccal mucosa. The preload and preload time were kept constant for all formulations. At the end of the preload time, the weight was removed from the pan and water was then added in the beaker in the left side pan through a burette at a constant rate until the bilayer tablet detached from the porcine buccal mucosa. The weight of the beaker containing water was noted as bioadhesive strength in grams [16].

### In Vitro Drug Release Study

*In vitro* drug release studies of the buccal tablets were conducted, in triplicate, on a simple standardized and modified apparatus. The dissolution apparatus consisted of a 250 ml beaker wrapped with a black polythene sheet as the receptor compartment; this was covered with a Perspex sheet with three holes, one for the temperature probe, the second for sampling, and the third for a donor tube. The donor tube was a glass rod attached with a grounded glass disk of 2 cm diameter. The backing layer side of the buccal tablet was attached at the bottom with the help of double adhesive foam tape. Before starting the study, the donor tube attached with the buccal tablet was introduced into the receptor compartment containing 100 ml of pre-warmed 37°C±1°C phosphate buffer of pH 6.8 in such a way that the tablet-releasing surface remained 2 cm below the surface of the buffer in the receptor compartment. The temperature was maintained at 37°C±1°C by a hot plate with a magnetic stirrer.

Dissolution fluid was stirred at a constant speed of 250 rpm using a Teflon-coated magnetic bead. Aliquots each of 1 ml were withdrawn at predetermined times with the help of pipettes and replaced with fresh buffer pre-warmed at 37°C±1°C temperature. All collected samples after proper dilutions were filtered through 0.45 µm filters and absorbance was measured at 255 nm using a UV Spectrophotometer (Shimadzu 1800, Japan). All solutions were protected from light till the absorbance was measured [17].

### Ex Vivo Permeation Study

The *ex vivo* permeation study of prochlorperazine maleate through porcine buccal mucosa was performed using a Franz diffusion cell at 37°C. Fresh porcine buccal mucosa was mounted between the donor and receptor compartments. The buccal tablet was placed with the core facing the mucosa and compartments clamped together. The donor compartment was filled with 1 ml phosphate buffer of pH 6.8. The receptor compartment (10 ml capacity) was filled with phosphate buffer of pH 6.8, and the hydrodynamics in the receptor compartment were maintained by stirring with a magnetic bead at 50 rpm. A 1 ml sample was withdrawn at predetermined time intervals and analyzed for drug content with suitable dilutions at 255 nm using a UV spectrophotometer [18].

## Authors’ Statements

### Competing Interests

The authors declare no conflict of interest.

### Animal Rights

Animal experiments were approved by the Institutional Animal Ethics Committee of Mohanlal Sukhadia University, Udaipur.

## References

[1] Harris D, Robinson JR (1992). Drug delivery via the mucous membranes of the oral cavity. J Pharm Sci.

[2] Darekar S, Khadabadi SS, Shahi SS (2014). Formulation and Evaluation of Bilayer buccal tablet of Sumatriptan succinate. Int J Pharm Pharm Sci.

[3] Patel AA, Patel K, Patel M, Patel N (2012). Formulation and Evaluation of Mucoadhesive buccal tablet of Vanlafaxine HCl. Am J Pharm Tech Res.

[4] Nagai T, Machida Y (1993). Buccal delivery systems using hydro-gels. Adv Drug Deliv Rev.

[5] Anders R, Merkle HP (1989). Evaluation of laminated mucoadhesive- patches for buccal drug delivery. Int J Pharm.

[6] Merkle HP, Wolany GJM, Duche'ne D (1996). Mucoadhesive patches for buccal peptide administration.

[7] Taylan B, Capan Y, Gu''ven O, Kes S, Hincal AA (1996). Design and evaluation of sustained released and buccal adhesive propranolol hydrochloride tablets. J Control Release.

[8] Li C, Bhatt PP, Johnston TP (1998). Evaluation of a mucoadhesive buccal patch for delivery of peptides: in vitro screening bioadhesion. Drug Dev Ind Pharm.

[9] Khoda Y, Kobayashi H, Baba Y, Yuasa H, Ozeki T, Kanaya Y, Sagara E (1997). Controlled release of lidocaine hydrochloride from buccal mucosaadhesive films with solid dispersion. Int J Pharm.

[10] Nozaki Y, Ohta M, Chien YW (1997). Transmucosal controlled systemic delivery of isosorbide dinitrate: in vivo/in vitro correlation. J Control Release.

[11] Nagasamy DV, Sankar S, Mayyanathan SN, Elango K (2010). Design and development of Prochlorperazine Maleate sustained release tablets: Influence of hydrophilic polymers on the release rate and in vitro evaluation. Int J Pharm Sci Nanotech.

[12] Shin SC, Kim JY (2000). Enhanced permeation of triamcinolone acetonide through the buccal mucosa. Eur J Pharm Biopharm.

[13] Aungst BJ, Rogers NJ (1989). Comparision of the effects of various transmucosal absorption promoters on buccal insulin delivery. Int J Pharm.

[14] Parodi B, Russo E, Caviglioli G, Cafaggi S, Bingardi G (1996). Development and characterization od a buccoadhesive dosage form of oxycodone hydrochloride. Drug Dev Ind Pharm.

[15] Bottenberg P, Cleymaet R, Muynek CD, Remon JP, Coomnas D, Slop D (1991). Development and testing of bioadhesive, fluoride containing slow release tablets for oral use. J Pharm Pharmacol.

[16] Gupta A, Garg S, Khar RK (1992). Measurement of bioadhesive strength of mucoadhesive buccal tablets: design of in vitro assembly. Indian Drugs.

[17] Agarwal V, Mishra B (1999). Design development and biopharmaceutical properties of buccoadhesive tablet. Drug Dev Ind Pharm.

[18] Pedersen M, Rassing MR (1991). Miconazole chewing gum as a drug delivery system test of release promoting additives. Drug Dev Ind Pharm.

